# Antioxidant Activities in Kenaf (*Hibiscus cannabinus*) Shoots during Growth Stages and Destination of Chlorogenic Acid and Kaempferol Glycosides

**DOI:** 10.3390/antiox13050532

**Published:** 2024-04-26

**Authors:** Shucheng Duan, Soon-Jae Kwon, Da Yun Jeong, Ji Hye Kim, You Rang Park, Chang Kyu Kim, Jae-Hee Kim, Seok Hyun Eom

**Affiliations:** 1 Graduate School of Green-Bio Science, Kyung Hee University, Yongin 17104, Republic of Korea; dsc97@khu.ac.kr (S.D.); jhkim96@khu.ac.kr (J.H.K.); yrspark@khu.ac.kr (Y.R.P.); kml8578@khu.ac.kr (C.K.K.); heeya@khu.ac.kr (J.-H.K.); 2 Advanced Radiation Technology Institute, Korea Atomic Energy Research Institute, Jeongeup 56212, Republic of Korea; soonjaekwon@kaeir.re.kr; 3 Department of Smart Farm Science, College of Life Sciences, Kyung Hee University, Yongin 17104, Republic of Korea; jeongdy3902@khu.ac.kr

**Keywords:** antioxidants, chlorogenic acid, kaempferol glycosides, kenaf, shoots, maturity stages

## Abstract

Apart from being utilized as a commercial fiber at maturity, kenaf shoots have potential as a food and feed source because of their diverse bioactivities. Previous studies have focused on mature stems because of their high biomass, whereas the antioxidant activities (AA) and the destination of AA contributors of kenaf stems and their high-yielding byproduct leaves during the growth stage have rarely been studied. Therefore, we investigated changes in AA and its relative components in kenaf leaves and stems during the four vital growth stages. Higher ABTS radical cation and DPPH radical scavenging abilities and ferric reducing antioxidant power, total phenolic content, total flavonoid content, and total polysaccharide content were observed at all leaf stages and in the late stem stages. Chlorogenic acid (CGA) and kaempferol glycosides, especially kaempferitrin (Kfr), were identified as representative phenolic acids and flavonoids in both kenaf leaves and stems. The content of CGA in both leaves and stems increased corresponding to the plant’s growth stage, whereas kaempferol glycosides were enhanced in leaves but declined in stems. The highest correlation was observed between TPC and AA in all organs. Further evaluation of CGA and Kfr verified that CGA was the predominant contributor to AA, surpassing Kfr. These findings suggest that kenaf leaves increase antioxidant levels as they grow and can be a useful source of stem harvesting byproducts.

## 1. Introduction

Antioxidants and antioxidant activities (AA) are vital factors in evaluating the nutritional values of edible plants. The intake of antioxidants from foods is closely related to human health, as it removes reactive oxygen species that the body produces during normal metabolic processes to alleviate oxidative damage [1]. Numerous studies have yielded significant results in evaluating the AA of many common vegetables and studying the contributors to their AA [2,3,4]. However, from the perspective of increasing access to a nutrient-rich and diverse food matrix, there are also many edible plants whose nutritional value, especially with respect to antioxidants, urgently needs to be studied. 

*Hibiscus cannabinus* (kenaf), an annual herbaceous crop, is famous worldwide for its high-quality fiber, mainly used, for example, in the paper and pulp, textiles, and manufacturing industries. Recently, increased attention has been paid to kenaf shoots utilization in both human food and animal feed production, not only because of their high yields, but also due to their health benefits [5]. The fresh leaves are usually eaten as vegetables after being cooked, and dried leaves are consumed as tea products or food additives [5,6,7]. Tender shoots are used as food, and the stalks are potential protein supplements for animal feed [5]. 

Variations in AA in kenaf across different organs and cultivars, and the use of solvents for extraction, have been extensively studied [8,9,10]. Ryu et al. [11] found that the DPPH radical scavenging ability and superoxide dismutase activity in kenaf leaves, seeds, and flowers exceeded that of stem bark across all solvent fractions. Birhanie et al. [12] observed significant variations in AA, as assessed by DPPH, ABTS, and ferric reducing antioxidant power (FRAP), among kenaf leaf extracts from 33 genotypes. Furthermore, phenolics are widely recognized as key contributors to AA in kenaf organs, particularly in leaves [12]. Pascoal et al. [13] identified that chlorogenic acids, and kaempferol and quercetin derivatives were major phenolics in kenaf leaves. Ryu et al. [14] reported kaempferol glycosides and chlorogenic acids as the primary phenolics identified in kenaf. Park et al. [15] showed that Kfr was the major phenolic compound in kenaf leaves, meanwhile chlorogenic acid (CGA) was the major phenolic compound in stems. However, the precise roles of these individual phenolic compounds in contributing to AA across various kenaf organs are still not fully understood. Additionally, while polysaccharides have been reported as vital contributors to the antioxidant capacity in kenaf leaves across various genotypes [12], their role in contributing AA in leaves and stems at different growth stages remains unclear. Thus, identifying high antioxidant sources within leaves and stems remains challenging, as the distribution of these antioxidants throughout the developmental stages has not been sufficiently clarified.

Considering the aforementioned information, kenaf plants were cultivated, and the leaves and stems were separately collected during four vital growth stages—young, fast, developmental, and ripening—to investigate the AA (DPPH assay, ABTS assay, and FRAP assay), total phenolic content (TPC), total flavonoid content (TFC), total polysaccharide content (TPSC), and individual phenolics among the organs. This study contributes to the determination of the appropriate harvest period for using kenaf shoots as a by-product source of antioxidants in the primary biomass production of fiber plants.

## 2. Materials and Methods

### 2.1. Chemicals and Solvents

HPLC-grade solvents, including water, methanol, and acetonitrile, were procured from Honeywell Company (Charlotte, NC, USA). Standard compounds of chlorogenic acid (≥95%) and kaempferitrin (>97%), were obtained from Sigma-Aldrich (Burlington, MA, USA). All other reagents used were of analytical grade.

### 2.2. Plant Materials

**Figure 1:**
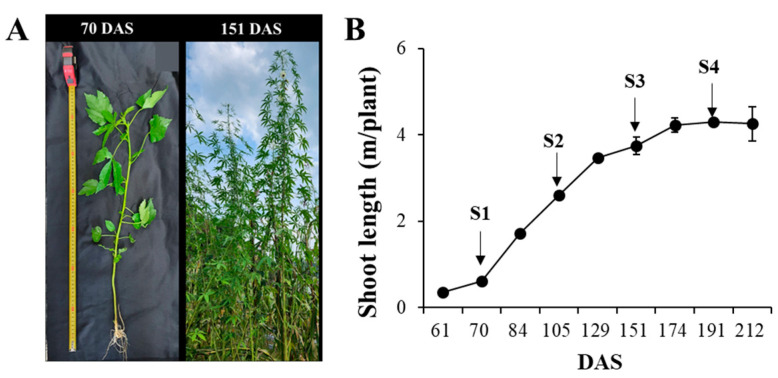
(**A**) Typical morphology changes of kenaf; and (**B**) variations in kenaf shoot length during life cycle. DAS indicates days after sowing.

Kenaf seeds originating in China were procured from a Korean market in 2022. After soaking for 24 h in distilled water, the seeds were planted in 72-cell seed trays filled with horticultural soil (Baroker; Seoulbio Co., Eumseong, Republic of Korea). The plants were adequately watered and cultivated in a greenhouse for 4 weeks. Subsequently, on 12 May, they were transplanted to an experimental field at Kyung Hee University (Yongin si, Republic of Korea). Samples were collected throughout the kenaf plant life cycle at 61, 70, 84, 105, 129, 151, 174, 191, and 212 days after sowing (DAS) in the soil. Each sampling event involved five individual plants and the growth pattern of each organ was recorded. The typical morphological changes and shoot length variation in the kenaf plants are shown in Figure 1. Considering the growth patterns and the observation that the plants reached the end of their life cycle after 212 DAS, four specific growth stages were identified for further analysis: 70, 105, 151, and 191 DAS for the young (S1), fast (S2), developmental (S3), and ripening (S4) stages, respectively. 

### 2.3. Drying Process

Kenaf organs were dried in a convective dry heat machine (Koencon Co., Ltd., Hanam, Republic of Korea) at temperatures <40 °C until a consistent weight was achieved. Subsequently, all the dried samples were ground and sieved through a 200-mesh sieve. 

### 2.4. Antioxidant Metabolites Measurement in Kenaf Organs at Different Growth Stages

An adaptation of the extraction method described by Ryu et al. [11] was used. One gram of the dried sample was immersed in 40 mL of 80% (*v*/*v*) aqueous methanol for 60 min with sonication at a temperature <40 °C. Subsequently, the samples were subjected to overnight extraction in a shaking incubator at 120 rpm and at a temperature <20 °C. Supernatants were obtained after centrifugation at 3000× *g* for 15 min.

#### 2.4.1. Determination of AA, Total Phenolic Content (TPC), Total Flavonoid Content (TFC), and Total Polysaccharide Content (TPSC)

Analyses of AA, TPC, and TFC were performed according to our previously reported methods [16], with some modifications. Roughly, AA were assessed using DPPH radical scavenging assay, ABTS radical cation scavenging assay, and FRAP assay, with radical scavenging abilities and reducing power expressed as mg of vitamin C equivalents (VCE)/g dry weight (d.w.). The Folin–Ciocalteu method and aluminum chloride colorimetric assay were used to determine the TPC and TFC, respectively. TPC results were expressed as mg of gallic acid equivalents (GAE)/g d.w., and TFC was expressed as mg of catechin equivalents (CE)/g d.w. The TPSC was determined using the phenol-sulfuric acid colorimetric assay. The prepared sample solution was diluted 50 times, and then 0.4 mL of this solution was combined with 0.2 mL of 5% phenol (*v*/*v*) and 1 mL of concentrated sulfuric acid. This mixture was incubated in a boiling water bath for 15 min. Following cooling, the absorbance of the sample was measured at 490 nm. The TPSC results were expressed as mg of glucose equivalents (GE)/g d.w.

#### 2.4.2. Phytochemical Analysis

##### HPLC Analysis of Phenolic Compounds

The phenolic compounds were analyzed as described by Pascoal et al. [13], with some modifications. After filtration through a 0.45-µm membrane filter, the samples were separated by gradient elution on a Prontosil 120-5-C18 SH column (250 × 4.6 mm, 5 μm, Bischoff, Leonberg, Germany) with a Waters 2695 Alliance HPLC (Waters Inc., Milford, MA, USA). Solvent A was 0.1% formic acid in water (*v*/*v*) and solvent B was 0.1% formic acid in acetonitrile. The following gradient was used for solvent B: 5% (0–0.5 min), 5–11% (0.5–3 min), 11–31% (3–40 min), 31–80% (40–42 min), 80% (42–45 min), 80–5% (45–46 min), 5% (46–48 min). An injection volume of 10 µL and a flow rate of 0.8 mL/min were used. A Waters 996 photodiode array detector (Waters, Inc.) was set at 280 nm. 

##### LC-MS/MS Analysis for Phenolics Identification 

The LC-MS/MS instrument used was an Agilent 6410 B (Agilent Technologies, Santa Clara, CA, USA). The LC-MS/MS conditions were a scanning mass range of *m*/*z* 126–800, both positive and negative ion sources, a gas temperature of 320 °C, gas flow of 35 L/min, and a capillary voltage of 4000 V. Both the auxiliary and sheath gases were nitrogen. The fragmentor voltage was 135 V. The collision energy was set at 30 eV. The Agilent 1200 HPLC conditions were an Epic C18 column (4.6 × 100 mm) (ES industries, West Berlin, NJ, USA), flow rate of 0.4 mL/min, injection volume of 1.0 µL, and a column temperature of 40 °C. The mobile phases used for LC were 0.1% formic acid in water (A) and 0.1% formic acid in acetonitrile (B). The retention time of the mobile phase was 95% (A) at 0–0.1 min, 35% (A) at 0.1–15 min, and 5% (A) at 15–20 min. 

##### Preparation of Standard Solutions and Quantification of Phenolic Compounds

The stock standard solutions of CGA and Kfr were prepared at a concentration of 500 μg/mL using 80% (*v*/*v*) aqueous methanol. Serial dilutions (1, 10, 20, 40 μg/mL) were performed on these stock solutions to prepare mixed standard working solutions. These solutions were subsequently analyzed by HPLC. The equation, correlation coefficient, LOD and LOQ for each phenolic standard are shown in Table S1. The quantification of CGA and kaempferol glycosides was calculated by the standard compounds CGA and Kfr, respectively.

### 2.5. Statistical Analysis

All data were collected in triplicate. Results are expressed as mean with SE. Analysis of variance was performed using SAS software (Enterprise Guide v. 8.3, SAS Institute Inc., Cary, NC, USA). Significant differences among samples were evaluated using ANOVA followed by Tukey’s HSD test, with a significance level of *p* < 0.05. Correlation analysis was carried out by calculating Pearson’s correlation coefficients between AA and various metabolite groups.

## 3. Results and Discussion

### 3.1. Variation of Fresh Weight, Dry Weight, and Water Content in Kenaf Organs

Table 1 illustrates the changes in fresh weight, dry weight, and water content of kenaf organs during different growth stages. The development of kenaf organs significantly enhanced the fresh weight and dry weight. Comparable amounts of fresh weight were observed between S3 and S4 in either leaves or stems. Similar results were also shown in dry weight changes in leaves, whereas this was gradually enhanced in stems. These results highlight the enhancement of dry matter content in kenaf stems during development. The water content of S1 leaves and stems were significantly higher than at late stages. The contents did not significantly change in leaves during development from S2 to S4, whereas this was gradually decreased in stems at S4 (Table 1). Leaves play a crucial role in various activities, such as photosynthesis, requiring a higher soluble content for nutrient transport and distribution in an aqueous environment. In contrast, stems primarily support the plant structure and absorb water and nutrients, leading to a higher proportion of insoluble substances that provide structural support and protection.

### 3.2. AA, TPC, TFC, and TPSC Changes in Kenaf Organs

**Figure 2:**
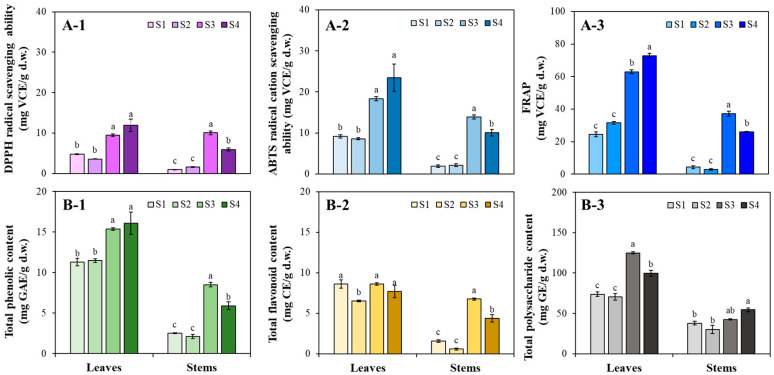
(**A-1**) DPPH radical scavenging ability; (**A-2**) ABTS radical cation scavenging ability; (**A-3**) ferric reducing antioxidant power (FRAP); (**B-1**) total phenolic content (TPC); (**B-2**) total flavonoid content (TPC); and (**B-3**) total polysaccharide content (TPSC) changes in kenaf organs at different growth stages (S1–S4). Different letters (a to c) indicate significant differences between each stage of the organs at *p* < 0.05 by Turkey’s HSD test.

**Figure 3:**
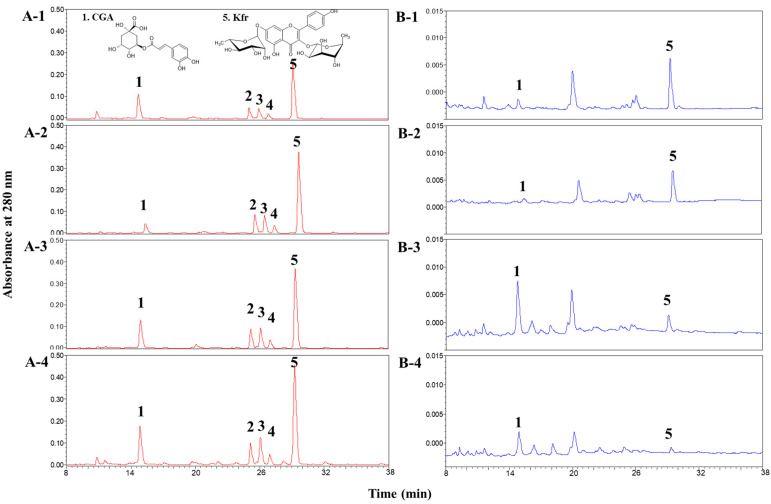
HPLC profiles of the extracts from various growth stages of (**A**) leaves; and (**B**) stems. The numbers (1 to 4) beside the capital letters indicate the stages of kenaf organs; S1 (**A-1**,**B-1**), S2 (**A-2**,**B-2**), S3(**A-3**,**B-3**), S4 (**A-4**,**B-4**). Number 1 to 5 indicate CGA, Kf-gly 1, Kf-gly 2, Kf-rham-xyl, and Kfr, respectively.

Figure 2 describes the variations in AA, TPC, TFC, and TPSC in kenaf organs at different growth stages. Regarding AA, similar variation patterns were observed in each kenaf organ between radical scavenging abilities (Figure 2(A-1,A-2)) and reducing power (Figure 2(A-3)), indicating that AA were significantly enhanced as development progressed, especially after S2. The leaves and stems exhibited higher AA at S4 (11.90 mg VCE/g d.w. of DPPH radical scavenging ability; 23.46 mg VCE/g d.w. of ABTS radical cation scavenging ability; 72.84 mg VCE/g d.w. of FRAP) and S3 (10.08 mg VCE/g d.w. of DPPH radical scavenging ability; 13.88 mg VCE/g d.w. of ABTS radical cation scavenging ability; 37.22 mg VCE/g d.w. of FRAP), respectively (Figure 2(A-1, A-2, and A-3)). TPC exhibited increasing patterns with the development of the various organs (Figure 2(B-1)). The leaves consistently showed relatively higher TPC (ranging from 11.27 to 16.07 mg GAE/g d.w.) compared to stems (ranging from 2.09 to 8.48 mg GAE/g d.w.), regardless of the stages. The variations in TFC significantly differed between leaves and stems (Figure 2(B-2)). Fluctuations in the TFC within the leaves were minimal across various growth stages, ranging from 6.50 to 8.60 mg CE/g d.w. The TFC in stems maintained low levels at the early stages (S1 and S2) and then significantly increased to the highest amount at S3, presenting 6.75 mg CE/g d.w. The leaves exhibited higher TPSC than stems across all stages, as shown in Figure 2B-3. Higher TPSC in leaves was observed in S4, presenting 124.95 mg GE/g d.w., while in stems, the higher content was observed in S3 (54.48 mg GE/g d.w.).

The positive relationship between AA and phenolic contents in various plant organs has been extensively documented [17,18,19,20]. However, previous studies on kenaf have typically focused on different organs harvested at the same stage or with the same organ collected from different genotypes of plants [8,11,12,21]. Thus, understanding the variation patterns of these valuable components—TPC, TFC, and AA—in leaves and stems throughout the kenaf life cycle remains challenging. Our results revealed a progressive pattern of increase in AA and TPC across the leaves and stems during different growth and developmental stages. Further, Pearson’s correlation analysis showed significant (*r* > 0.95; *p* < 0.001) positive correlations between TPC, TFC, and AA in various kenaf organs, except for the relatively weak correlations (*r* > 0.70; *p* < 0.05) between TFC and AA in the leaves. Similar results were also reported by Birhanie et al. [12], who investigated the correlation between AA with TPC, TFC, and polysaccharide content in kenaf leaves across 33 genotypes. Their findings indicated that TPC and polysaccharide content significantly contributed to AA, encompassing ferric reducing antioxidant potential and DPPH radical and ABTS radical cation scavenging abilities, whereas TFC did not. However, in our study, the correlations between TPSC and AA were not significantly observed in leaves (*r* < 0.5; *p* > 0.05). The distinct correlation patterns between AA and TPSC in kenaf leaves strongly suggest that polysaccharides were less affected by AA, while phenolics were the dominant antioxidants in kenaf leaves during the developmental stages. Strong correlation coefficients between AA and TPSC in stems were shown in both ABTS (*r* = 0.600, *p* < 0.05) and FRAP (*r* = 0.598, *p* < 0.05) assays. The different effects of TPSC to AA between kenaf leaves and stems may be attributed to differences in polysaccharide compositions and contents across common plant tissues [22]. Based on our results, it can be concluded that kenaf mature shoots are potentially valuable sources of antioxidant ingredients, owing to the high accumulation of phenolics. Kenaf leaves are excellent sources of antioxidant phenolics, especially after the developmental stage (S3), and exhibit stronger AA than the stems at all stages. Different plant species with various organs have exhibited distinct influences on the accumulation of phenolics during development, exhibiting complex patterns including increasing, decreasing, maintaining, and fluctuating [23,24,25,26] patterns. However, since the specific changes in phenolic content in kenaf leaves and stems during plant development have still remained unclear, our results indicate that phenolic content in both kenaf leaves and stems increases during plant growth and contributes to the enhancement of AA.

### 3.3. Phenolics Variations in Kenaf Shoots

To elucidate the contributors to AA in kenaf shoots, individual phenolic compounds were further identified and quantified. The identified phenolic compounds in kenaf shoots are shown in Table 2. The peak numbers (1 to 5) correspond to the peaks, as depicted in Figure 3. The identification of peak 1 and 5 was conducted by comparing the retention time and maximum absorbance wavelengths (λmax) with those of authentic standards. Other peaks (2–4) were considered to be kaempferol derivatives, as their λmax values consisted of 264.8 nm, presenting the characteristics of kaempferol aglycone. Peaks 2 and 3 were considered to be kaempferol glycoside 1 (Kf-gly 1) and kaempferol glycoside 2 (Kf-gly 2), respectively, based on the interpretation of their MW or MS/MS spectra and those provided in the literature [13]. Peak 4 was considered to be a kaempferol rhamnosyl xyloside (Kf-rham-xyl), based on the interpretation of the MW 564.2 and information reported in the literature. The fragment with *m*/*z* 433.1 was considered to be a kaempferol with a rhamnose substitution. In addition, the fragment with *m*/*z* 287.1 was found to correspond to the kaempferol core in peaks 3–5. The MS/MS spectra of individual phenolics are displayed in Figure S1. At each kenaf leaf stage, five major phenolics: one phenolic acid (CGA) and four kaempferol glycosides—Kf-gly 1, Kf-gly 2, Kf-rham-xyl, and Kfr were identified. Meanwhile, CGA and Kfr were identified at different growth stages of the stems. Figure 3 shows the chemical structures of CGA and Kfr found in both leaves and stems, along with the HPLC profiles of extracts from various growth stages of the leaves and stems.

#### 3.3.1. Phenolics Variations in Kenaf Leaves

In the leaves, a gradual increase in each phenolic content was observed (Table 3). This trend aligns with the patterns observed in the changes of TPC (Figure 2(B-1)). The CGA content was significantly lower during the early growth stages (2.43 mg/g d.w. at S1, 1.84 mg/g d.w. at S2) compared to the later growth stages (5.41 mg/g d.w. at S3, 6.01 mg/g d.w. at S4). Kfr accounted for more than 50% of the total content among the detected phenolics, regardless of the stage. Similar variation patterns were observed in kaempferol glycosides during kenaf development, with the lowest content of each kaempferol glycoside at S1 and non-significant variations at other periods. 

Chlorogenic acids, as well as kaempferol and quercetin derivatives, were identified as the major phenolic compounds in the methanolic extracts of two cultivars of kenaf leaves [13]. Park et al. [15] reported that CGA and Kfr are representative phenolic acids and flavonoids, respectively, in the leaves of two kenaf cultivars. Ryu et al. [11] studied the phytochemical constituents of kenaf leaves in various solvents and found that Kfr was the major flavonoid. In contrast, caffeic acid was the major phenolic compound, followed by chlorogenic acid. These results suggest the different phytochemical compositions of kenaf leaves among varieties. However, previous studies have not clarified the phenolic variation patterns among the kenaf developmental stages. Our study revealed the gradual enhancement of each phenolic content in the leaves as the plants matured (after S3). This indicates that leaves can be harvested simultaneously with stems during maturation, serving as a valuable source of phenolics, rather than being discarded as waste. 

#### 3.3.2. Phenolics Variations in Kenaf Stems

Regarding stems, a pattern of initially increasing and then decreasing content was observed in CGA, which reached its peak at 0.38 mg/g d.w. during S3. In contrast, Kfr exhibited a gradual decrease in content during stem development, with values of 0.57 mg/g d.w. at S1, 0.41 mg/g d.w. at S2, 0.19 mg/g d.w. at S3, and 0.09 mg/g d.w. at S4. Previous research by Park et al. [15] reported CGA as the major phenolic acid in the stems of two kenaf cultivars—‘Jangdae’ and ‘Jeokbong’—with no detection of Kfr. Conversely, Ryu et al. [11] found that Kfr and caffeic acid were the dominant phenolics in kenaf bark, with CGA being absent in various solvents. These results highlight the variability in the composition of phenolic compounds in kenaf stems. However, there are few reports about changes in the main phenolic compounds in stems in the plant growth cycle. Kenaf stems are usually harvested at maturity to obtain high-quality fiber [10]. Our results showed reverse accumulation patterns between CGA and Kfr in the stems during plant growth. The content of CGA was more abundant in the later stages of stems (S3 and S4) than in the early stages, while that of Kfr was more abundant in the early stages (S1 and S2) than in the later stages.

### 3.4. Antioxidant Activity of Individual Phenolics

CGA and kaempferol glycosides (especially, Kfr) were identified as representative phenolic acids and flavonoids in both kenaf leaves and stems (Table 3). However, Pearson’s correlation analysis showed that both leaves and stems exhibited low positive correlation coefficients (r < 0.52, *p* > 0.05 of leaves; *r* < −0.78, *p* < 0.01 of stems) between kaempferol glycoside(s) and AA, compared to the strong positive correlation (r > 0.66, *p* < 0.05 of leaves; r > 0.95, *p* < 0.001 of stems) between CGA and AA (Tables S2 and S3). This observation led to the tentative speculation that AA in the leaves and stems of kenaf originated primarily from phenolic acids, particularly CGA, rather than the more prominently abundant Kfr. To validate this assumption, we conducted further evaluations of AA in standard samples of CGA and Kfr using widely recognized antioxidants, vitamin C (Vc) and Kf (the aglycone of Kfr), as reference materials.

**Figure 4:**
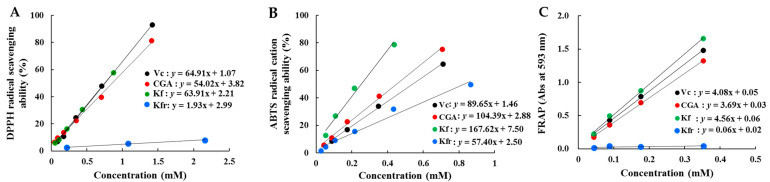
(**A**) DPPH radical scavenging ability; (**B**) ABTS radical cation scavenging ability; and (**C**) FRAP in standard CGA, Kf, Kfr, and Vc.

Figure 4 shows a comparison of the AA from the four standard samples. Vc, Kf, and CGA demonstrated similar DPPH radical scavenging activities and FRAP, which were obviously stronger than Kfr (Figure 4A,C). Kf showed the highest ABTS radical cation scavenging ability, followed by CGA, Vc, and Kfr (Figure 4B). Overall, CGA demonstrated a markedly higher antioxidant capacity compared to Kfr, with scavenging abilities 33-fold and 1.9-fold greater for DPPH radicals and ABTS radical cations, respectively. Moreover, the reducing power of CGA was 61.5-fold greater than Kfr. The differences in AA displayed by the two substances across various analytical methods can be attributed to the differences in their reaction mechanisms [27]. In the DPPH assay, DPPH serves as a unique lipophilic radical that can be transformed into its reduced forms (DPPH-H or DPPH). In contrast, the ABTS assay is ideal for assessing antioxidant components possessing hydrophilic or lipophilic properties. This assay relies solely on a hydrogen atom transfer reaction. The FRAP assay demonstrates the antioxidant’s ability to reduce ferric ions. Based on the aforementioned results, it is evident that the representative phenolic acid (CGA) and flavonoid (Kfr) are key antioxidant contributors in kenaf leaves. Furthermore, these phenolics significantly contributed to the high correlation between to TPC and AA. However, the observed relatively weak correlation between TFC and AA in the leaves can be ascribed to Kfr, as evidenced in Table S2, since it comprises a significant portion of the content yet possesses comparatively lower radical scavenging capabilities (Table 3; Figure 4). In the case of stems, CGA emerges as one of the primary contributors to AA. Not only is the AA from Kfr low, but the content of Kfr also shows a negative pattern with AA. Additionally, Kf, the aglycone form of Kfr, demonstrates strong AA. 

## 4. Conclusions

This study investigated the AA, TPC, TFC, TPSC and individual phenolic compounds in kenaf leaves and stems at four vital growth stages. The leaves consistently exhibited higher AA than the stems. Leaves at all stages, as well as stems in the later growth stages, emerged as excellent sources of TPC, TFC, and TPSC. Moreover, in both the leaves and stems, CGA and Kfr were identified as the predominant phenolic acids and flavonoids, respectively. Further examination of the AA in these standards revealed that CGA was the primary contributor to AA in both stems and leaves, despite the abundance of Kfr. Additionally, Kf—the aglycone of Kfr—exhibited stronger AA than Kfr. Overall, the stems and leaves of kenaf are good sources of antioxidant substances, but their contents varied significantly between organs, and this was influenced by growth stages. Antioxidants accumulated significantly at the ripening stage in all organs, with leaves showing significantly higher AA than stems. Considering that kenaf cultivation primarily aims to obtain mature stems for fiber production, there is potential for the industrial harvesting of leaves and stems to obtain antioxidants for human food or animal feed applications. 

## Figures and Tables

**Table 1:** Fresh weight, dry weight, and water content in kenaf shoots at different growth stages.

Stages	Leaves			Stems		
	Fresh Weight (g/Plant)	Dry Weight (g/Plant)	Water Content (%)	Fresh Weight (g/Plant)	Dry Weight (g/Plant)	Water Content (%)
S1	18 ± 1 c	0.8 ± 0.2 c	95 ± 0 a	16.3 ± 0.2 c	0.5 ± 0.0 d	97 ± 0 a
S2	315 ± 34 b	64 ± 10 b	80 ± 3 b	566 ± 16 b	130 ± 12 c	77 ± 3 b
S3	568 ± 81 a	114 ± 17 a	80 ± 5 b	1314 ± 138 a	263 ± 37 b	80 ± 5 b
S4	417 ± 51 a	102 ± 11 a	76 ± 5 b	1408 ± 117 a	486 ± 55 a	65 ± 4 c

Different letters (a to d) indicate significant differences between each stage of the organs at *p* < 0.05 by Turkey’s HSD test.

**Table 2:** Phenolic compounds identified in kenaf shoots by HPLC and LC-MS/MS.

Peak No.	Rt (min)	MW	*m*/*z ^+^*	MS/MS ^b^	λmax (nm) ^c^	Identification
1	14.92	354.3			216.5/325.5	CGA ^d^
2 ^a^	25.20	594.2	593.2	238.9/160.9	264.8/345.8	Kf-gly 1
3	26.12	594.3	595.3	433.2/287.1	264.8/347.0	Kf-gly 2
4	26.99	564.2	565.2	433.1/287.1	264.8/345.8	Kf-rham-xyl
5	29.31	578.5	579.2	433.2/287.1	263.7/342.2	Kfr

^a^ The identification was conducted at negative ion mode. ^b^ The major fragments were shown. ^c^ The results of the λmax were obtained by HPLC. ^d^ The identification of CGA was conducted by HPLC using a standard compound. Abbreviation: CGA, chlorogenic acid; Kf, kaempferol; rham-xyl, rhamnosyl xyloside; Kfr, kaempferitrin. The *m/z ^+^* was obtained at positive ion mode.

**Table 3:** Quantitative changes in phenolic compounds (mg/g d.w.) at different stages of kenaf leaves and stems.

Organs	Stages	CGA	Kf-gly 1	Kf-gly 2	Kf-rham-xyl	Kfr
Leaf	S1	2.43 ± 0.04 b	3.16 ± 0.03 b	3.31 ± 0.03 b	1.47 ± 0.01 b	17.90 ± 0.23 b
	S2	1.84 ± 0.02 c	5.59 ± 0.06 a	5.62 ± 0.08 ab	2.35 ± 0.04 ab	26.90 ± 0.42 ab
	S3	5.41 ± 0.07 a	5.87 ± 0.12 a	6.88 ± 0.16 a	2.79 ± 0.06 a	27.30 ± 0.62 ab
	S4	6.01 ± 0.82 a	5.24 ± 0.95 ab	6.95 ± 1.20 a	2.49 ± 0.42 a	28.13 ± 4.10 a
Stem	S1	0.07 ± 0.00 c	N.D	N.D	N.D	0.57 ± 0.04 a
	S2	0.04 ± 0.00 c	N.D	N.D	N.D	0.41 ± 0.01 b
	S3	0.38 ± 0.00 a	N.D	N.D	N.D	0.19 ± 0.00 c
	S4	0.19 ± 0.02 b	N.D	N.D	N.D	0.09 ± 0.01 c

The quantification of CGA and kaempferol glycosides were calculated by the standard compounds CGA and Kfr, respectively. N.D. indicates not detected. Different letters (a to c) indicate a significant difference between each stage of leaf at *p* < 0.05 by Turkey’s HSD test.

## Data Availability

Data are contained within the article.

## Supplementary Materials

The following supporting information can be downloaded at: https://www.mdpi.com/article/10.3390/antiox13050532/s1, Table S1: The equation, correlation coefficient, LOD and LOQ for each phenolic standard, Figure S1: LC-MS/MS chromatograms of kenaf extracts, Table S2: Correlation coefficients between antioxidant activities and metabolite groups assembled with kenaf leaves.
