# Supplementary material for: Antioxidant Activities in Kenaf (Hibiscus cannabinus) Shoots during Growth Stages and Destination of Chlorogenic Acid and Kaempferol Glycosides
Source: Antioxidants (Basel). 2024 Apr 26;13(5):532. doi: 10.3390/antiox13050532 (PMC11117515; doi:10.3390/antiox13050532)
Supplement: Supplementary file 1 [file antioxidants-13-00532-s001.zip › antioxidants-2932251-supplementary.pdf]

Table S1. The equation, correlation coefficient, LOD and LOQ for each phenolic standard

Phenolics	Equation	R^2	LOD (μg/ml)	LOQ (μg/ml)
CGA	y=17278x-8771.6	0.9987	3.79	11.47
Kfr	y=10424x-4417.7	0.999	3.45	10.45

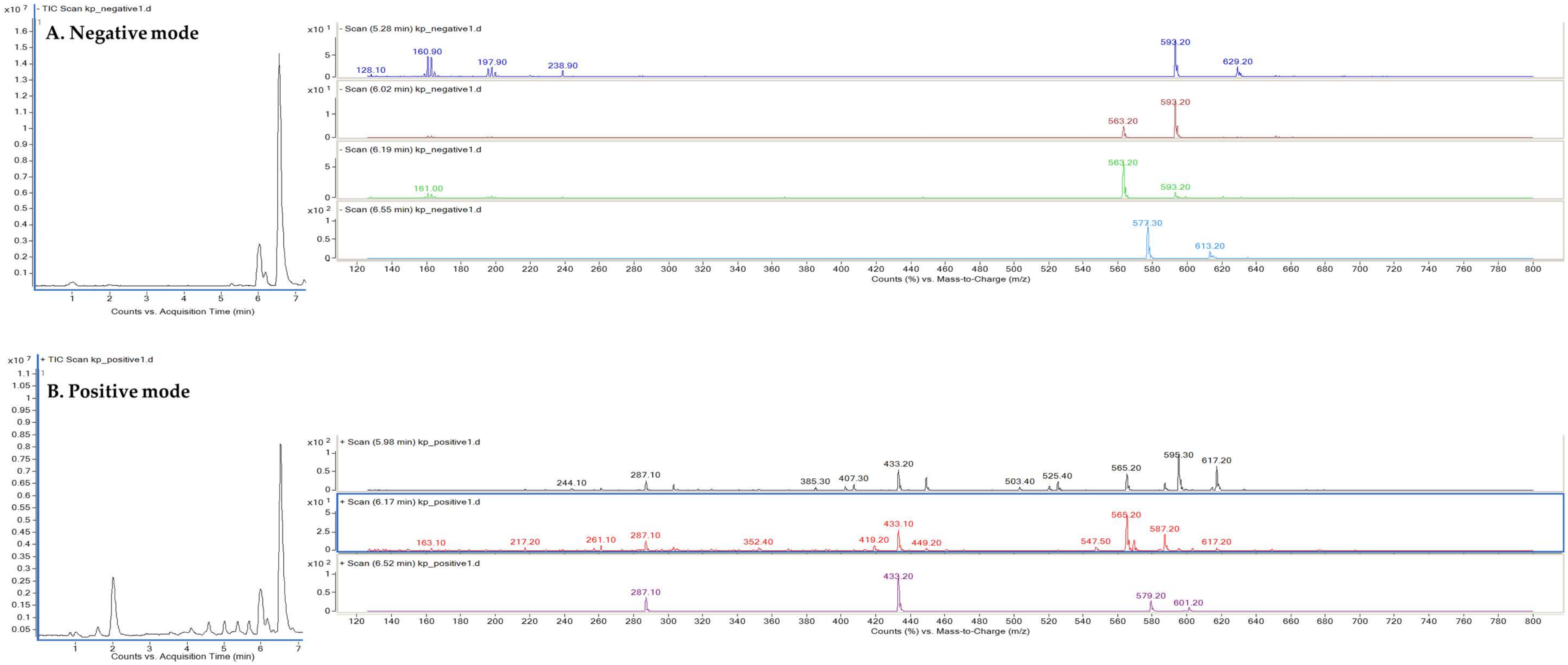

Figure S1. LC-MS/MS chromatograms of kenaf extracts.

Table S2. Correlation coefficients between antioxidant activities and metabolite groups assembled with kenaf leaves.

	CGA	Kf-gly 1	Kf-gly 2	Kf-rham-xyl	Kfr	TFC	TPC	TPSC	DPPH	ABTS	FRAP
CGA	1										
Kf-gly 1	0.168ns	1									
Kf-gly 2	0.480ns	0.929***	1								
Kf-rham-xyl	0.361ns	0.976***	0.974***	1							
Kfr	0.281ns	0.968***	0.966***	0.959***	1						
TFC	0.821**	-0.234ns	0.080ns	-0.024ns	-0.162ns	1					
TPC	0.598*	0.278ns	0.526ns	0.425ns	0.341ns	0.700*	1				
TPSC	0.522ns	-0.294ns	0.013ns	-0.199ns	-0.056ns	0.432ns	0.283ns	1			
DPPH	0.733**	0.202ns	0.507ns	0.367ns	0.306ns	0.767**	0.960***	0.473ns	1		
ABTS	0.665*	0.231ns	0.520ns	0.380ns	0.334ns	0.705*	0.971***	0.452ns	0.992***	1	
FRAP	0.693*	0.481ns	0.736**	0.623*	0.566ns	0.596*	0.908***	0.322ns	0.924***	0.923***	1

Tukey's HSD test, * indicates $p < 0.05$, ** indicates $p < 0.01$, *** indicates $p < 0.001$, and ns indicates no significance.

Table S3. Correlation coefficients between antioxidant activities and metabolite groups assembled with kenaf stems.

	CGA	Kfr	TFC	TPC	TPSC	DPPH	ABTS	FRAP
CGA	1							
Kfr	-0.6753*	1						
TFC	0.981***	-0.738**	1					
TPC	0.980***	-0.767**	0.993***	1				
TPSC	0.451ns	-0.655*	0.575ns	0.541ns	1			
DPPH	0.980***	-0.784**	0.970***	0.986***	0.484ns	1		
ABTS	0.958***	-0.833***	0.979***	0.991***	0.600*	0.985***	1	
FRAP	0.960***	-0.819**	0.972***	0.977***	0.598*	0.976***	0.984***	1

Tukey's HSD test, * indicates $p < 0.05$, ** indicates $p < 0.01$, and *** indicates $p < 0.001$.
